# Nutritional and inflammatory biomarkers in predicting spontaneous anastomotic leakage closure following enterocutaneous fistula resection: the role of postoperative CRP-lymphocyte ratio

**DOI:** 10.3389/fnut.2025.1631484

**Published:** 2025-12-11

**Authors:** Tianchi Yu, Xiaofeng Liao, Shikun Luo, Xin Xu, Risheng Zhao, Yunzhao Zhao, Zheng Yao

**Affiliations:** 1 Interventional Department, Jiangning Hospital, Nanjing, Jiangsu, China; 2 Department of General Surgery, Jiangning Hospital, Nanjing, Jiangsu, China

**Keywords:** postoperative, fistula, surgery, outcomes, spontaneous closure

## Abstract

**Background:**

To determine the correlation between the C-reactive protein-lymphocyte ratio (CLR) and the spontaneous resolution of anastomotic leakage (AL) subsequent to the resection of enterocutaneous fistulas (ECF).

**Methods:**

In this retrospective cohort analysis, we examined adult patients who experienced AL following ECF resection from January 2019 to January 2024. Observations continued for 90 days post-surgery, during which CLR values were recorded at designated intervals. The relationship between CLR and the spontaneous resolution of leakage by 30 and 90 days was evaluated using logistic regression and restricted cubic splines.

**Results:**

Out of 107 participants, 35 (32.7%) exhibited spontaneous resolution of leakage within 30 days, while 90 (84.1%) did so by the 90-day mark. A significant association was noted between CLR on day 7 post-leakage and 30-day resolution (OR = 0.97, 95%CI: 0.94–0.99, *p* = 0.03). Additionally, CLR on day 14 post-leakage correlated with 90-day resolution (OR = 0.88, 95%CI: 0.79–0.95, *p* = 0.003). A J-shaped curve was observed in the relationship between CLR levels and closure rates at both time frames. Notably, the resolution rate was considerably lower in patients whose ECF was attributable to pancreatitis, with an OR of 0.21 (*p* = 0.02) relative to other causes.

**Conclusion:**

Decreasing CLR subsequent to leakage may be one of supportive predictor for spontaneous closure of AL following ECF resection.

## 1 Introduction

Enterocutaneous fistulas (ECFs) are abnormal communications between the gastrointestinal tract and the skin, posing a significant clinical challenge with mortality rates ranging from 6 to 33% (1–3). Management of ECFs typically follows a stepwise approach: initial stabilization, control of sepsis, optimization of nutritional status, and finally, delayed surgical intervention when the patient is clinically suitable (4). Nutritional rehabilitation is considered essential during the conservative phase, as malnutrition is common among ECF patients due to prolonged inflammation, protein loss, and inadequate oral intake (4, 5).

Definitive management—fistula resection and anastomosis—may be delayed for several weeks or months to allow for adequate nutritional repletion and resolution of intra-abdominal inflammation. However, the success of this surgical procedure varies widely and is influenced by factors such as the severity of intra-abdominal adhesions, patient comorbidities, and the quality of postoperative care. One of the most feared complications following ECF resection is anastomotic leakage (AL), which can lead to prolonged hospitalization, increased morbidity, and elevated mortality rates (6–8).

Importantly, spontaneous closure of AL is possible and has been reported in over 80% of cases in experienced centers, particularly when optimal infection control and comprehensive nutritional support are provided (9). These observations underscore the critical role of host immune-nutritional status in postoperative tissue healing.

Recent studies have highlighted the C-reactive protein (CRP)-lymphocyte ratio (CLR) as a potential prognostic biomarker across a range of surgical and inflammatory conditions (10–12). CLR integrates two fundamental aspects of recovery: the systemic inflammatory burden (reflected by CRP) and immunonutritional competence (reflected by circulating lymphocyte counts) (10, 13). As such, CLR may serve as a surrogate marker for the body’s capacity to resolve inflammation and support tissue regeneration following major surgery.

In the context of ECF surgery, dynamic changes in CLR after infection control may provide valuable insights into the patient’s readiness for recovery and the likelihood of spontaneous AL closure. A significant postoperative reduction in CLR could indicate a restored immunonutritional balance conducive to wound healing. Conversely, persistently elevated CLR levels may suggest ongoing inflammation or nutritional insufficiency, identifying patients at higher risk of poor healing outcomes.

Therefore, we hypothesize that a marked decline in CLR following infection control is associated with a higher probability of spontaneous AL resolution after ECF resection. This study aims to evaluate postoperative CLR as a predictive biomarker of spontaneous leak closure, potentially enabling clinicians to personalize postoperative nutritional strategies and avoid unnecessary reoperations in select patients.

## 2 Materials and methods

### 2.1 Patients and outcomes

This was a retrospective cohort study conducted at a tertiary center. Adult patients who experienced recurrent AL following ECF resection from January 2019 to January 2024 were included. Patients were followed for up to 90 days post-resection. The primary outcome was the rate of spontaneous closure within 30 days post-leakage. The secondary outcome assessed spontaneous closure within 90 days post-leakage, following a treatment period. The influence of postoperative CLR at various time points was analyzed.

### 2.2 ECF surgery

Definitive surgery for ECF was postponed until patients had been infection-free for at least 1 month and met the following criteria: absence of active tumors, Body Mass Index (BMI) of ≥18.0 kg/m^2^, adequate physical recovery, hemoglobin ≥100 g/L, albumin ≥30 g/L, and a minimum of 3 months following fistula diagnosis (9). If recurrent AL occurred, the criteria for subsequent ECF surgery remained identical to those for the initial surgery.

### 2.3 Definition of spontaneous closure

Contrast imaging was performed upon cessation of intestinal fluid drainage. During the procedure, iodixanol was injected via the drainage tube under fluoroscopic guidance. Non-visualization of contrast extravasation indicated spontaneous closure.

### 2.4 Postoperative CRP-albumin-lymphocyte index

Postoperative laboratory examinations were performed every other day following ECF resection. CLR was calculated as follows: CRP (mg/L)/absolute lymphocyte count (cells/μL) × 10^3^. AL was diagnosed through contrast gastrointestinal imaging when prompted by intestinal fluid discharge from the drainage tube. Subsequent CT scan was conducted to assess postoperative abdominal infections. Antibiotic therapy was discontinued when the patient’s procalcitonin level normalized and no abdominal fluid accumulation was present, regardless of leakage status. If abdominal effusion was detected by CT, percutaneous drainage was performed for source control. Follow-up CT was conducted 48 h later to evaluate drainage effectiveness. Antibiotics were discontinued once fluid accumulation resolved procalcitonin normalized.

When (1) no abdominal fluid accumulation was observed by CT post-leakage and (2) normal procalcitonin levels were recorded on the day of leakage diagnosis, CLR was defined as the value on the day of leakage. For patients with abdominal infections following leakage, CLR was evaluated once patients were free from abdominal fluid accumulation and elevated procalcitonin levels, and was recorded as the CLR on the day of leakage.

### 2.5 Data collection and statistical analysis

Statistical analyses were performed using IBM SPSS Statistics version 26.0 (IBM Analytics, Armonk, NY) and R version 4.3.2 (R Foundation for Statistical Computing). The Mann–Whitney U and Kruskal–Wallis tests were used for continuous variables, while Fisher’s exact test was applied to categorical variables. Postoperative CLR on days 1, 7, and 14 post-leakage was examined. Logistic regression was used to assess associations with clinical indices (including demographic characteristics, preoperative characteristics, intraoperative characteristics, postoperative characteristics, and comorbidities) after adjusting for potential confounders. The relationship between CLR and spontaneous closure within 30 and 90 days post-leakage was analyzed using restricted cubic splines with three knots at the 10th, 50th, and 90th percentiles to provide optimal flexibility while maintaining model stability and avoiding overfitting. Statistical significance was set at *p* < 0.05.

## 3 Results

### 3.1 Population and characteristics

A total of 421 patients underwent ECF surgical resection at our center, with AL occurring in 107 (25.4%) cases. The median age of enrolled patients was 48 years (IQR: 42–55 years), and the median BMI was 20.7 kg/m^2^ (IQR: 19.6–22.6 kg/m^2^). Males comprised 59.8% (*n* = 64) of the enrolled population. The etiologies of ECF included trauma (*n* = 43), tumors (*n* = 11), pancreatitis (*n* = 34), and intestinal obstruction from previous surgeries (*n* = 19). The fistulas were located in the small bowel (*n* = 44), ileocolic anastomosis (*n* = 9), and colon (*n* = 54). Thirty-five (32.7%) patients achieved spontaneous closure within 30 days post-leakage. In total, 90 (84.1%) patients experienced spontaneous closure within 90 days (Table 1).

**Table 1:** Baseline characteristics of enrolled patients.

Variables	Total (*n* = 107)
Demography	
Male, No. (%)	64 (59.8)
Age, year; (median, IQR)	48 (42–55)
Preoperative BMI, kg/m2, (median, IQR)	20.7 (19.6–22.6)
Characteristics of ECF	
Interval from occurrence to excision of ECF, weeks, (median, IQR)	16 (13–18)
High output, No. (%)	15 (14)
Requiring parenteral nutrition, No. (%)	7 (6.5)
Etiology of ECF, No. (%)	
Trauma	43 (40.1)
Tumor	11 (10.3)
Pancreatitis	34 (31.8)
Intestinal obstruction due to previous surgery	19 (17.8)
Location of ECF, No. (%)	
Small bowel	44 (41.1)
Ileocolic anastomosis	8 (7.5)
Colon	55 (51.4)
Preoperative characteristics of ECF excision	
Preoperative albumin, g/L, (median, IQR)	37 (34–40)
Preoperative hemoglobin, g/L, (median, IQR)	120 (112–132)
Preoperative WBC, 10^9^/L, (median, IQR)	6.3 (4.4–7.7)
Preoperative CRP, mg/dL, (median, IQR)	11.3 (7.4–15.1)
Intraoperative characteristics of ECF excision	
Adhesion degree*, (median, IQR)	4 (4–5)
Bleeding during excision, ml, (median, IQR)	1,400 (1100–1,600)
Duration of excision, min, (median, IQR)	270 (230–310)
Postoperative characteristics of excision	
WBC on the day of leakage, 10^9^/L, (median, IQR)	9.2 (7.8–10.9)
Prealbumin on the day of leakage, mg/dL	22.4 (18.9–28.2)
Albumin on the day of leakage, g/L	32.4 (31.2–34.3)
The amount of red blood cells infused during and within48 h after excision**, U, (median, IQR)	7 (5–8)
The amount of albumin infused during and within48 h after excision***, g (median, IQR)	60 (50–80)
Requring surgical intervention after postoperative abdominal infections following the leakage, No. (%)	14 (9.3)
Puncture drainage	14(9.3)
Laparotomy	0
Duration from excision to anastomotic leakage detected, day, (median, IQR)	8 (7–10)
Comorbidity, No. (%)	
Hypertension	3 (2.8)
Impaired glucose tolerance in postoperative period****	21 (19.6)

*According to Tian et al. (5), the abdominal adhesions were classified into the following five grades: Grade I = no adhesions; Grade II = minimal adhesions localized to one or two areas; Grade III = diffuse adhesions, but not extensive; Grade IV = diffuse extensive adhesions, easily lysed; Grade V = diffuse extensive, dense adhesions, difficult to lyse. **In order to maintain the Hemoglobin >100 g/L within 48 hours after surgery. ***In order to maintain the Albumin >30 g/L within 48 hours after surgery. **** Insulin is needed to control postoperative blood glucose levels below 10 mmol/L. BMI, Body Mass Index; CRP, C-reactive protein; ECF, Enterocutaneous fistulas; WBC, white blood cell.

### 3.2 Infections following AL

AL typically occurred on the 8th day post-resection (IQR: 7th - 10th day). Fourteen patients underwent puncture drainage for abdominal infections following leakage. Due to the source control effects of the double-lumen irrigation-suction tube, abdominal infection was absent in the remaining 93 patients when leakage was detected. Among the 14 patients with abdominal infection, after the effective puncture and drainage, the duration of the infection was 9 days (IQR: 6–10 days) after puncture and drainage.

### 3.3 C-reactive protein-lymphocyte ratio

Initially, the CLR post-leakage was measured at 140 (IQR: 122–163), with a median CRP of 103 mg/L (IQR: 84–129 mg/L) and lymphocyte count of 0.8 × 10^9^ (IQR: 0.6–0.9 × 10^9^/L)., By 7 days post-diagnosis, CLR had decreased to 59 (IQR: 46–71), with a median CRP of 86 mg/L (IQR: 64–111 mg/L) and lymphocyte count of 1.3 × 10^9^/L (IQR: 1.2–1.8 × 10^9^/L). Fourteen days post-leakage, the CLR further declined to 18 (IQR:14–24), with a median CRP of 28 mg/L (IQR: 21 - 39 mg/L) and lymphocyte count of 1.7 × 109 (IQR: 1.3–2.2 × 109/L). Differences in CLR between patients achieving spontaneous closure within 30 or 90 days are presented in Supplementary Table 1. Additionally, CLR values were comparable on the day of leakage, 7 days post-diagnosis, and 14 days post-leakage across different leakage locations (Supplementary Table 2) and different etiologies (Supplementary Table 3).

### 3.4 Association of CLR and spontaneous closure

**Figure 1:**
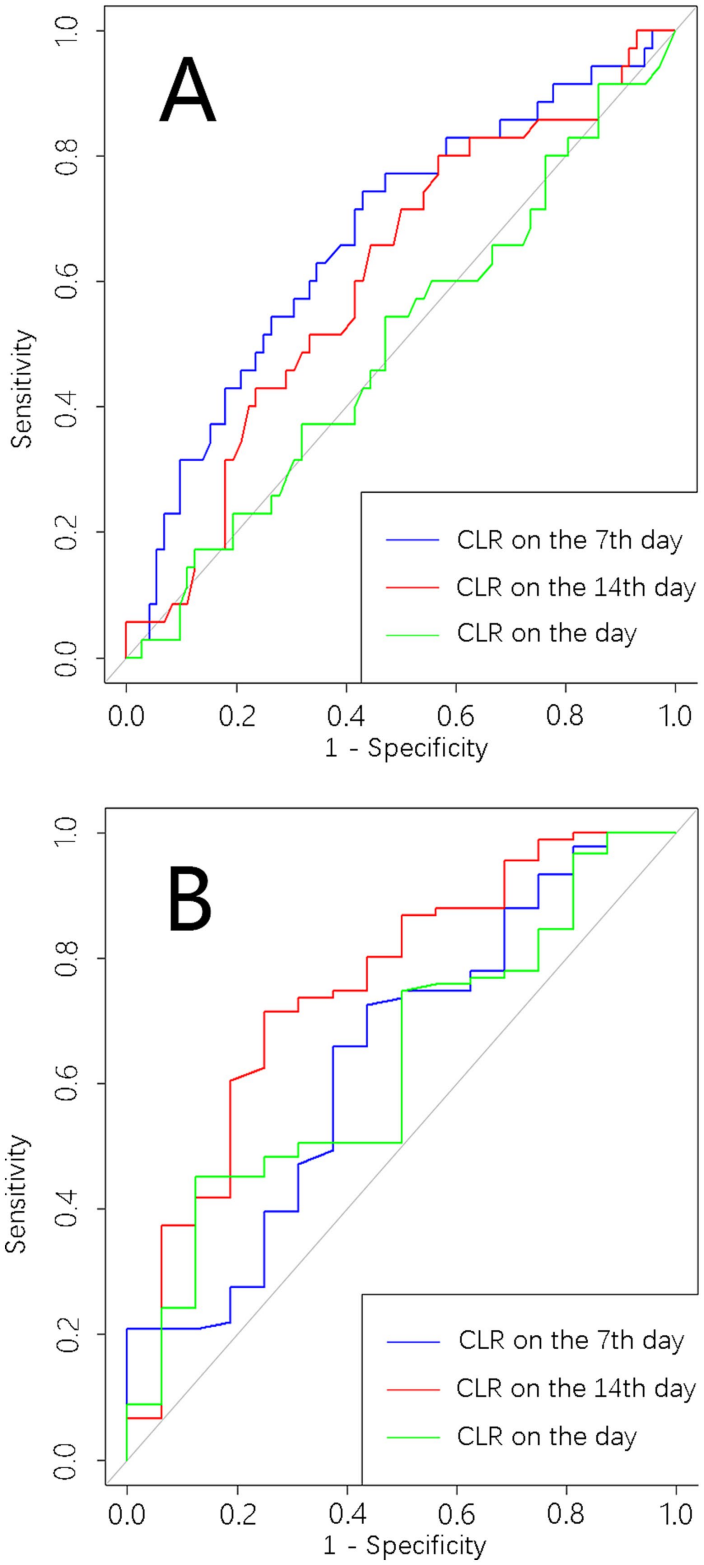
Predictive performance for spontaneous closure after leakage. **(A)** Receiver-operating characteristic (ROC) analysis for spontaneous closure within 30 days of CLR at different time points. **(B)** ROC analysis for spontaneous closure within 90 days of CLR at different time points.

**Figure 2:**
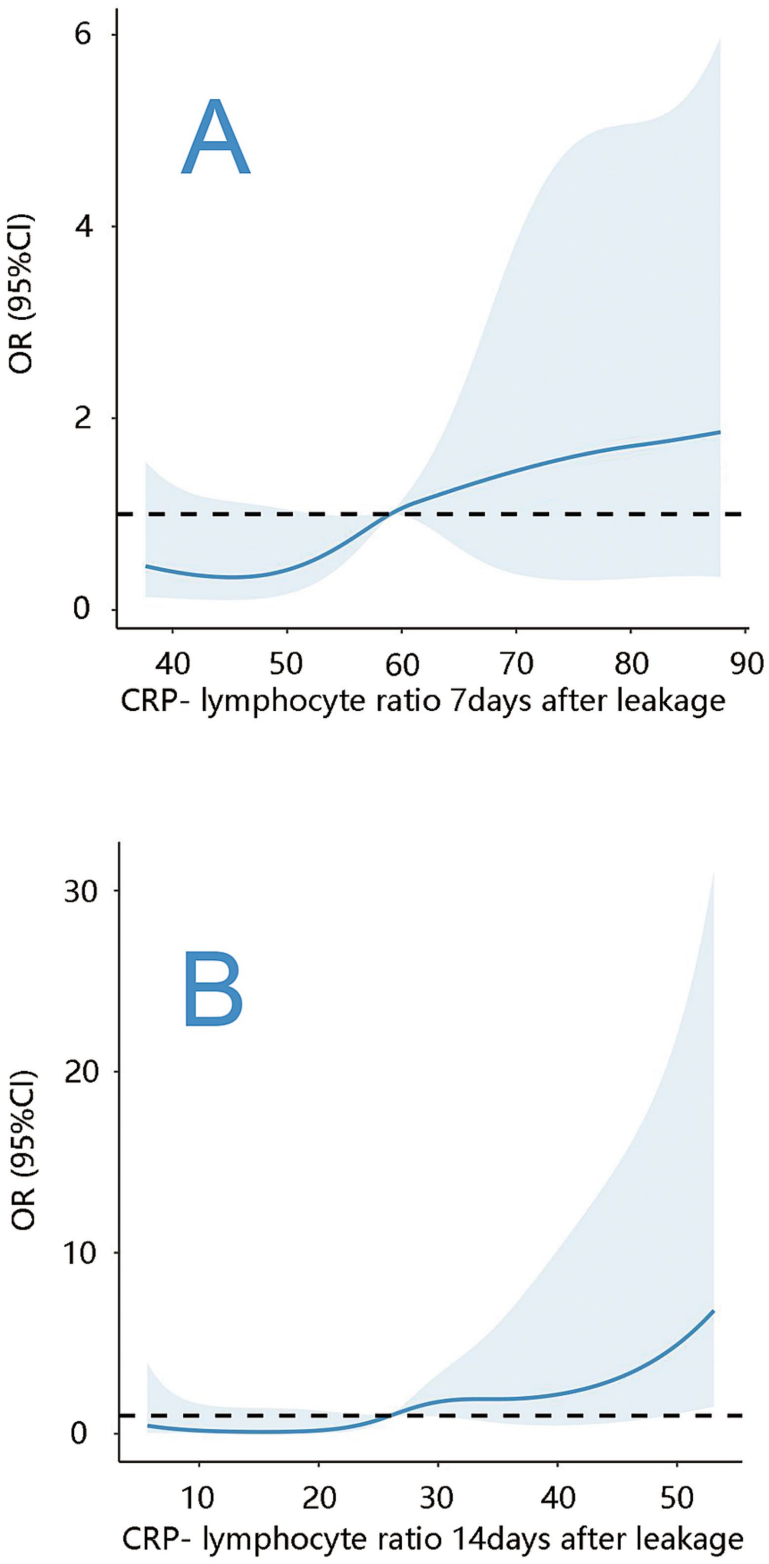
Association of CLR with spontaneous closure after leakage. **(A)** Association of CLR measured 7 days after leakage with spontaneous closure. **(B)** Association of CLR measured 14 days after leakage with spontaneous closure.

Univariate analysis revealed significant differences in CLR on the seventh day post-leakage, incidence of required surgical intervention after postoperative abdominal infections, and etiology of ECF between patients with and without spontaneous closure within 30 days (Table 2). Multivariate analysis showed that CLR on the seventh day post-leakage (OR = 0.97, 95%CI: 0.94–0.99, *p* = 0.03) was predictive of spontaneous closure within this period. Pancreatitis as an etiology was associated with a reduced closure rate (OR = 0.21, 95%CI: 0.06–0.75, *p* = 0.02, Table 3) compared to trauma as the reference. The AUC of CLR on Day 1, Day 7, and Day 14 were 0.50 (95% CI, 0.39–0.76, *p* = 0.96), 0.67 (95% CI, 0.57–0.79, *p* = 0.002), and 0.61 (95% CI, 0.49–0.72, *p* = 0.06), respectively (Figure 1A), for evaluating spontaneous closure within 30 days post-leakage. The CLR cutoff was 58.1 with 60.6% sensitivity, and 59.6% specificity. A J-shaped association between CLR on the seventh day post-leakage and spontaneous closure was observed (Figure 2A).

**Table 2:** Univariate analysis for spontaneous closure within 30 days.

Variables	Non spontaneous closure (*n* = 72)	Spontaneous closure (*n* = 35)	*p*
Male, No. (%)	45 (62.5)	19 (54.3)	0.42
Age, year; (median, IQR)	48 (44–55)	47 (35–58)	0.35
Preoperative BMI, kg/m2, (median, IQR)	20.5 (19.4–22.5)	21.2 (20.0–22.9)	0.29
Interval from occurrence to excision of ECF, weeks, (median, IQR)	16 (13–18)	15 (14–18)	0.47
High output, No. (%)	10 (13.9)	5 (14.3)	0.96
Requiring parenteral nutrition, No. (%)	4 (5.6)	3 (8.6)	0.55
Etiology of ECF, No. (%)			0.01
Trauma	27 (37.5)	16 (45.7)	
Tumor	5 (6.9)	6 (17.1)	
Pancreatitis	30 (41.6)	4 (11.4)	
Intestinal obstruction due to previous surgery	10 (13.9)	9 (25.7)	
Location of ECF, No. (%)			0.11
Small bowel	26 (36.1)	18 (51.5)	
Ileocolic anastomosis	4 (5.6)	4 (11.4)	
Colon	42 (58.3)	13 (37.1)	
Preoperative albumin, g/L, (median, IQR)	37 (33–40)	38 (34–41)	0.63
Preoperative hemoglobin, g/L, (median, IQR)	119 (112–131)	120 (117–138)	0.89
Preoperative WBC, 10^9^/L, (median, IQR)	6.3 (4.5–7.6)	6.3 (4.3–7.8)	0.45
Preoperative CRP, mg/dL, (median, IQR)	11.5 (7.8–15.3)	10.7 (6.6–14.5)	0.20
Preoperative CRP-lymphocyte ratio, (median, IQR)	7 (7–9)	7 (6–9)	0.36
Adhesion degree, (median, IQR)	4 (4–5)	4 (4–5)	0.82
Bleeding during excision, ml, (median, IQR)	1,500 (1100–1700)	1,400 (1100–1,600)	0.46
Duration of excision, min, (median, IQR)	270 (240–310)	270 (230–300)	0.33
WBC on the day of leakage, 10^9^/L, (median, IQR)	9.3 (7.8–11.1)	9.1 (7.4–10.6)	0.67
Prealbumin on the day of leakage, g/L	22.1 (18.4–28.1)	24.1 (19.5–28.4)	0.21
Albumin on the day of leakage, g/L	32.2 (31.1–34.1)	32.8 (31.9–34.7)	0.49
The amount of red blood cells infused during and within48 hours after excision, U, (median, IQR)	7 (5–8)	7 (5–8)	0.96
The amount of albumin infused during and within48 hours after excision, g (median, IQR)	60 (50–80)	60 (40–80)	0.80
Requring surgical intervention after postoperative abdominal infections following the leakage, No (%)	13 (18.1)	1 (2.9)	0.03
Puncture drainage	13 (18.1)	1 (2.9)	
Laparotomy	0	0	
Duration from excision to anastomotic leakage detected, day, (median, IQR)	7 (7–9)	8 (7–10)	0.25
CRP-lymphocyte ratio on the day of leakage, (median, IQR)	140 (119–163)	141 (122–164)	0.96
CRP-lymphocyte ratio seven days after leakage, (median, IQR)	61 (50–71)	53 (44–64)	0.02
CRP-lymphocyte ratio 14 days after leakage, (median, IQR)	19 (15–24)	17 (13–20)	0.10
Hypertension	2 (2.8)	1 (2.9)	0.99
Impaired glucose tolerance in postoperative period	15 (20.8)	6 (17.1)	0.65

BMI, Body Mass Index; CRP, C-reactive protein; ECF, Enterocutaneous fistulas; WBC, white blood cell.

**Table 3:** Multivariate analysis for spontaneous closure within 30 days.

Variables	OR	95% CI	*p*
CLR on the 7th day after leakage	0.97	0.94–0.99	0.03
Etiology of ECF			
Trauma	Ref		
Tumor	4.03	0.82–19.53	0.09
Pancreatitis	0.21	0.06–0.75	0.02
Intestinal obstruction due to previous surgery	1.35	0.43–4.22	0.61
Requring surgical intervention after postoperative abdominal infections following the leakage	0.15	0.015–1.45	0.10

CLR, C-reactive protein -lymphocyte ratio; ECF, Enterocutaneous fistulas.

Statistical differences in CLR on the seventh and fourteenth day post-leakage, etiology, and location were noted between patients with or without spontaneous closure within 90 days post-leakage (Table 4). Multivariate analysis indicated that CLR on the fourteenth day post-leakage (OR = 0.88, 95%CI: 0.79–0.95, *p* = 0.003, Table 5) was predictive of spontaneous closure within this period. The AUC of CLR on Day 1, Day 7, and Day 14 were 0.63 (95% CI, 0.49–0.77, *p* = 0.07), 0.64 (95% CI, 0.49–0.79, *p* = 0.07), and 0.75 (95% CI, 0.62–0.89, *p* < 0.001), respectively (Figure 1B), for evaluating spontaneous closure within 90 days post-leakage. The cutoff was 19.8 with 75% sensitivity and 67.1% specificity. A J-shaped association between CLR on the fourteenth day post-leakage and spontaneous closure was observed (Figure 2B).

**Table 4:** Univariate analysis for spontaneous closure within 90 days.

Variables	Non spontaneous closure (*n* = 16)	Spontaneous closure (*n* = 91)	*p*
Male, No. (%)	10 (62.5)	54 (59.3)	0.69
Age, year; (median, IQR)	49 (49–59)	48 (41–55)	0.33
Preoperative BMI, kg/m2, (median, IQR)	20.8 (19.8–23.1)	20.7 (19.5–22.6)	0.57
Interval from occurrence to excision of ECF, weeks, (median, IQR)	17 (14–18)	16 (13–18)	0.41
High output, No. (%)	2 (12.5)	13 (14.3)	0.85
Requiring parenteral nutrition, No. (%)	2 (12.5)	5 (5.5)	0.29
Etiology of ECF, No. (%)			0.03
Trauma	4 (25)	39 (42.9)	
Tumor	1 (6.3)	10 (10.9)	
Pancreatitis	10 (62.5)	24 (26.3)	
Intestinal obstruction due to previous surgery	1 (6.3)	18 (19.8)	
Location of ECF, No. (%)			0.04
Small bowel	2 (12.5)	42 (46.2)	
Ileocolic anastomosis	2 (12.5)	6 (6.6)	
Colon	12 (75)	43 (47.2)	
Preoperative albumin, g/L, (median, IQR)	35 (32–39)	37 (34–41)	0.61
Preoperative hemoglobin, g/L, (median, IQR)	118 (110–134)	121 (112–132)	0.80
Preoperative WBC, 10^9^/L, (median, IQR)	6.4 (4.8–7.6)	6.2 (4.3–7.8)	0.36
Preoperative CRP, mg/dL, (median, IQR)	11.6 (7.9–14.5)	11.3 (7.3–15.2)	0.68
Adhesion degree, (median, IQR)	5 (4–5)	4 (4–5)	0.21
Bleeding during excision, ml, (median, IQR)	1,500 (1000–1800)	1,400 (1100–1,600)	0.44
Duration of excision, min, (median, IQR)	280 (220–330)	270 (230–310)	0.46
WBC on the day of leakage, 10^9^/L, (median, IQR)	9.1 (7.7–10.6)	9.2 (7.8–10.9)	0.75
Prealbumin on the day of leakage, g/L	22.2 (19.0–27.5)	22.4 (18.9–28.2)	0.59
Albumin on the day of leakage, g/L	32.3 (30.6–33.6)	32.4 (31.3–34.3)	0.29
The amount of red blood cells infused during and within48 hours after excision, U, (median, IQR)	7 (5–8)	8 (5–8)	0.38
The amount of albumin infused during and within48 hours after excision, g (median, IQR)	70 (60–80)	60 (50–80)	0.71
Requring surgical intervention after postoperative abdominal infections following the leakage, No. (%)	4 (25)	10 (10.9)	0.13
Puncture drainage	4 (25)	10 (10.9)	
Laparotomy	0	0	
Duration from resction to anastomotic leakage detected, day, (median, IQR)	8 (7–10)	8 (7–10)	0.76
CLR on the day of leakage, (median, IQR)	149 (135–175)	140 (119–163)	0.21
CLR on the 7th day after leakage, (median, IQR)	67 (51–82)	58 (46–70)	0.05
CLR on the 14th day after leakage, (median, IQR)	23 (20–30)	18 (12–23)	0.001
Hypertension	1 (6.3)	2 (2.2)	0.38
Impaired glucose tolerance in postoperative period	4 (25)	17 (18.7)	0.56

BMI, Body Mass Index; CLR, C-reactive protein -lymphocyte ratio; CRP, C-reactive protein; ECF, Enterocutaneous fistulas; WBC, white blood cell.

**Table 5:** Multivariate analysis for spontaneous closure within 90 days.

Variables	OR	95% CI	*p*
Location of ECF			
Small bowel	Ref		
Ileocolic anastomosis	0.19	0.01–2.64	0.15
Colon	0.17	0.02–1.98	0.19
CLR on the 7th days after leakage	0.97	0.94–1.01	0.22
CLR on the 14th day after leakage	0.88	0.79–0.95	0.003
Etiology of ECF			
Trauma	Ref		
Tumor	2.11	0.15–29.53	0.58
Pancreatitis	0.18	0.03–0.87	0.03
Intestinal obstruction due to previous surgery	0.68	0.03–13.45	0.80

CLR, C-reactive protein -lymphocyte ratio; ECF, Enterocutaneous fistulas.

## 4 Discussion

The results of this study provide insight into the role of CLR in predicting the spontaneous closure of AL after ECF resection. Significant reduction in CLR, particularly on the 7th and 14th days following AL diagnosis, was associated with higher probabilities of spontaneous closure within 30 and 90 days, respectively. This finding supports the hypothesis that reduced postoperative inflammatory states, indicated by lower CLRs, facilitate anastomotic site healing and enhance spontaneous closure.

CRP, a well-established acute-phase protein produced by the liver in response to inflammation, signals active inflammatory processes commonly observed in postoperative patients. Previous research has emphasized the importance of CRP in postoperative outcomes. For instance, McKechnie et al. (14) observed that lower postoperative CRP levels were associated with improved outcomes in colorectal surgery patients. Similarly, Ren et al. (15) found that higher postoperative CRP levels were associated with recurrent fistula following ECF resection. Lymphocytes, essential for adaptive immunity and tissue repair, play pivotal roles in host cell-mediated defense against infections and tumors (16). Additionally, peripheral lymphocyte count is regarded as a significant nutritional indicator in patients with various diseases (17). Therefore, CLR acts as a marker of the balance between the inflammatory response (indicated by CRP levels) and immune competence (evaluated through lymphocyte counts). High CLR suggests an intensified inflammatory state with possibly compromised immune response, while decreased CLR indicates reduced inflammatory burden and enhanced immune capabilities, both essential for effective anastomotic site healing. Notably, our study revealed a distinctive J-shaped association between CLR and spontaneous closure probability, representing a novel and clinically significant finding that challenges the traditional linear understanding of inflammatory markers and healing outcomes. This J-shaped curve indicates that both extremely low and extremely high CLR values are associated with reduced spontaneous closure rates, while moderate CLR levels correlate with optimal healing potential. The identification of this non-linear relationship is particularly important as it suggests that excessively suppressed inflammatory responses may be as detrimental to healing as excessive inflammation, highlighting the need for balanced immune-inflammatory status in successful wound healing.

The integration of CLR into postoperative decision-making protocols could significantly enhance the management of patients with recurrent AL following ECF resection. Our findings suggest that CLR measurements on the seventh day post-leakage for 30-day outcomes and fourteenth day for 90-day outcomes could serve as valuable biomarkers to guide clinical interventions. Specifically, persistently elevated CLR values may prompt intensified nutritional support strategies, including optimization of protein intake and micronutrient supplementation to support lymphocyte recovery and reduce systemic inflammation. Additionally, CLR trends could inform the timing of potential reoperation decisions—patients with declining CLR trajectories may benefit from conservative management with continued monitoring, while those with persistently high or rising CLR values might require earlier consideration for surgical intervention. The observed J-shaped association between CLR and spontaneous closure rates suggests that optimal CLR thresholds could be established to stratify patients into risk categories, thereby facilitating personalized treatment algorithms. Furthermore, CLR monitoring could complement traditional clinical assessments in determining appropriate duration of antibiotic therapy and timing of nutritional interventions, ultimately contributing to more precise and individualized patient care pathways.

Supporting these findings, previous studies have demonstrated the prognostic significance of CLR in various surgical contexts. For example, a systematic review and meta-analysis by Ye et al. (18) showed that a higher preoperative CLR was associated with poorer outcomes in patients undergoing gastrointestinal surgery. Similarly, Wang et al. (19) found that a higher CLR predicted worse prognosis in patients with colorectal cancer. Okugawa et al. (20) indicated that the assessment of preoperative lymphocytes and CRP could help physicians evaluate surgical and oncological risks in gastric cancer patients. As our understanding of recurrent fistula following ECF resection deepens, it has become evident that higher inflammation levels correlate with lower likelihood of spontaneous closure (8, 9). Our study appears to be the first to comprehensively analyze inflammation and immunity using CLR. Other inflammatory markers, such as the neutrophil-to-lymphocyte ratio (NLR) and platelet-to-lymphocyte ratio (PLR), have also been investigated (21, 22). However, neutrophil level variation after ECF resection is not as significant as that of CRP, making CRP a more reliable indicator of postoperative inflammation (7–9). ECF surgery for recurrent fistula often involves significant intraoperative and postoperative bleeding, leading to platelet loss. While platelets can indicate inflammation severity, they are not as indicative as CRP in this context (7, 8).

The findings of this study contribute to the growing body of evidence highlighting the potential utility of CLR as a marker for monitoring healing process and predicting the likelihood of spontaneous closure following ECF resection. We also observed that underlying etiology of ECF affects the spontaneous closure rate, with patients experiencing ECF due to pancreatitis having a lower rate of spontaneous closure compared to those with trauma-induced ECF. This observation is consistent with previous studies that found ECF following pancreatitis is associated with mesangial injury, which impairs blood supply and adversely affects spontaneous closure. Intestinal fistula caused by pancreatitis presents significant challenges, as it often results from mesenteric corrosion by pancreatitis, where infection directly penetrates from the retroperitoneum into the peritoneum through the mesentery. This means that during intestinal fistula surgery, mesenteric anatomy is already disrupted or compromised, making surgery extremely difficult. Additionally, necrotizing pancreatitis leading to intestinal fistula often creates potential scar cavities in the retroperitoneum and mesenteric base. Forceful dissection of these cavities can cause greater tissue damage. Furthermore, these cavities constitute components of the purulent cavity wall in necrotizing pancreatitis (although obvious purulence may not be present during definitive intestinal fistula surgery), and these purulent cavity walls may contribute to postoperative stress responses, causing aggravated intra-abdominal inflammation that adversely affects anastomotic site healing.

Despite these significant findings, our study has several limitations. First, the retrospective design may introduce selection bias and limit result generalizability. Future prospective studies are required to validate our findings and establish causal relationships. Second, this single-center study may have limited external validity. Multi-center studies would provide more comprehensive data and enhance finding applicability. Third, the absence of long-term follow-up data represents a significant limitation. We acknowledge that many delayed intestinal fistulas may occur several months after surgery, and initially closed fistulas may develop recurrent symptoms months later. Therefore, lack of extended follow-up may have missed such patients, and future research should incorporate comprehensive long-term monitoring. Fourth, comprehensive inflammatory biomarker data, particularly cytokines such as IL-6 and TNF-*α*, were not systematically collected across all patients. Under our institutional protocol, inflammatory markers are measured only in patients with suspected or confirmed infections, while non-infected patients do not routinely undergo such assessments. This selective approach prevented establishment of correlations between specific inflammatory mediators and fistula closure outcomes, which could have provided stronger mechanistic evidence for the relationship between inflammation and fistula healing. Moreover, while CLR is a promising marker, it is influenced by various factors including infection, stress, and comorbidities, which may confound results. Future studies should adjust for these potential confounders to provide more accurate prognostic information.

It is crucial to emphasize that CLR should not be considered a standalone predictor but rather a complementary biomarker to be integrated with clinical assessment, imaging findings, and other laboratory parameters in a comprehensive decision-making framework. Most importantly, this study represents a single-center retrospective analysis without external validation, which significantly limits the generalizability of our findings. Additionally, while CLR provides clinically meaningful objective information, it should not be used in isolation but rather as part of a comprehensive assessment strategy. Future research should focus on developing integrated prediction models that combine CLR with other established factors such as fistula characteristics, nutritional parameters, and additional inflammatory markers to enhance predictive accuracy and clinical utility. Such multiparameter approaches may overcome limitations of single biomarker prediction and provide more robust tools for ECF management. Infection management may potentially influence CRP and lymphocyte levels. When infection is effectively resolved, it facilitates spontaneous fistula closure, and CLR may have minor influence on this process. The association between CLR and fistula closure may reflect the state of infection control during follow-up.

Finally, this research focused on short-term outcomes, specifically at 30 and 90 days. Comprehensive long-term follow-up is essential to thoroughly evaluate the persistent effects of CLR on surgical outcomes and to detect any delayed complications or AL recurrences.

## 5 Conclusion

Significant reductions in CLR, observed on the 7th and 14th days following leakage, may serve as a supportive predictor of enhanced spontaneous closure probability at 30 and 90 days post-leakage. However, CLR should be interpreted as a contributory factor rather than a determinative indicator and used in conjunction with other clinical parameters for optimal prognostic assessment.

## Data Availability

The raw data supporting the conclusions of this article will be made available by the authors, without undue reservation.
