# Supplementary material for: Nutritional and inflammatory biomarkers in predicting spontaneous anastomotic leakage closure following enterocutaneous fistula resection: the role of postoperative CRP-lymphocyte ratio
Source: Front Nutr. 2025 Dec 11;12:1631484. doi: 10.3389/fnut.2025.1631484 (PMC12739880; doi:10.3389/fnut.2025.1631484)
Supplement: Supplementary file 1 [file Table_1.doc]

Supplemental Table 1. The differences of the data on CRP-lymphocyte ratio between patients achieving spontaneous closure within 30days or 90days

|  | Total | Within 30 days | | | Within 90 days | | |
| --- | --- | --- | --- | --- | --- | --- | --- |
|  |  | None spontaneous closure (n=72) | Spontaneous closure (n=35) | *P* | None spontaneous closure (n=16) | Spontaneous closure (n=91) | *P* |
| CRP-lymphocyte ratio on the day of leakage,(median,IQR) | 140 (122 - 163) | 140 (122 - 164) | 141 (119 - 163) | 0.96 | 149 (135 - 175) | 140 (119 - 163) | 0.21 |
| CRP,mg/L (median,IQR) | 104 (84 - 130) | 104 (86 - 132) | 99 (74 - 126) | 0.25 | 110 (81 - 141) | 101 (83 - 128) | 0.29 |
| Lymphocyte,*10^9^ (median,IQR) | 0.7 (0.6 - 0.9) | 0.7 (0.6 - 0.9) | 0.8 (0.7 - 0.9) | 0.24 | 0.6 (0.5 - 0.8) | 0.7 (0.6 - 0.9) | 0.09 |
| CRP-lymphocyte ratio seven days after leakage, (median,IQR) | 59 (46 - 71) | 61 (50 - 71) | 53 (44 - 64) | 0.02 | 67 (51 - 82) | 58 (46 - 70) | 0.05 |
| CRP,mg/L (median,IQR) | 86 (64 - 111) | 87 (65 - 109) | 72 (58 - 136) | 0.01 | 97 (63 - 121) | 85 (64 - 109) | 0.06 |
| Lymphocyte,*10^9^ (median,IQR) | 1.3 (1.2 - 1.8) | 1.3 (1.1 - 1.7) | 1.6 (1.2 - 2.1) | 0.07 | 1.2 (1.1 - 1.6) | 1.3 (1.2 - 1.8) | 0.10 |
| CRP-lymphocyte ratio 14 days after leakage,(median,IQR) | 18 (14 - 24) | 19 (15 - 24) | 17 (13 - 20) | 0.10 | 23 (20 - 30) | 18 (12 - 23) | 0.001 |
| CRP,mg/L (median,IQR) | 29 (21 - 43) | 30 (22 - 44) | 25 (19 - 36) | 0.05 | 45 (24 - 60) | 28 (20 - 37) | 0.03 |
| Lymphocyte,*10^9^ (median,IQR) | 1.7 (1.3 - 2.2) | 1.7 (1.4 - 2.3) | 1.8 (1.3 - 2.2) | 0.77 | 1.7 (1.2 - 2.1) | 1.7 (1.4 - 2.2) | 0.57 |
