# Supplementary material for: Nutritional and inflammatory biomarkers in predicting spontaneous anastomotic leakage closure following enterocutaneous fistula resection: the role of postoperative CRP-lymphocyte ratio
Source: Front Nutr. 2025 Dec 11;12:1631484. doi: 10.3389/fnut.2025.1631484 (PMC12739880; doi:10.3389/fnut.2025.1631484)
Supplement: Supplementary file 2 [file Table_2.doc]

Supplemental Table 2. CRP-lymphocyte ratio at different location and different time

|  | Small bowel | Ileocolic anastomosis | Colon | *P* |
| --- | --- | --- | --- | --- |
| CRP-lymphocyte ratio on the day of leakage,(median,IQR) | 145 (123 - 164) | 153 (141 - 184) | 135 (116 - 164) | 0.12 |
| CRP-lymphocyte ratio seven days after leakage, (median,IQR) | 59 (46 - 70) | 61 (52 - 81) | 59 (46 - 68) | 0.30 |
| CRP-lymphocyte ratio 14 days after leakage,(median,IQR) | 18 (14 - 23) | 18 (12 - 23) | 18 (13 - 23) | 0.94 |
