# Supplementary material for: Nutritional and inflammatory biomarkers in predicting spontaneous anastomotic leakage closure following enterocutaneous fistula resection: the role of postoperative CRP-lymphocyte ratio
Source: Front Nutr. 2025 Dec 11;12:1631484. doi: 10.3389/fnut.2025.1631484 (PMC12739880; doi:10.3389/fnut.2025.1631484)
Supplement: Supplementary file 3 [file Table_3.doc]

Supplemental Table 3. CRP-lymphocyte ratio at different etiologies.

|  | Trauma | Tumor | Pancreatitis | Obstruction due to previous surgery | *P* |
| --- | --- | --- | --- | --- | --- |
| CRP-lymphocyte ratio on the day of leakage,(median,IQR) | 143 (125 - 163) | 144 (133 - 167) | 132 (113 - 158) | 141 (119 - 161) | 0.51 |
| CRP-lymphocyte ratio seven days after leakage, (median,IQR) | 59 (47 - 67) | 61 (55 - 65) | 59 (46 - 74) | 57 (45 - 68) | 0.64 |
| CRP-lymphocyte ratio 14 days after leakage,(median,IQR) | 18 (14 - 23) | 17 (8 - 22) | 19 (14 - 26) | 17 (14 - 23) | 0.52 |
